# Supplementary material for: Mapping the Quantitative Dose–Response Relationships Between Nutrients and Health Outcomes to Inform Food Risk–Benefit Assessment
Source: Foods. 2025 Apr 20;14(8):1420. doi: 10.3390/foods14081420 (PMC12026834; doi:10.3390/foods14081420)
Supplement: Supplementary file 1 [file foods-14-01420-s001.zip › foods-3580410-supplementary.pdf]

Supplementary Table S1. Search strategy and result counts for each included nutrient and database.

Nutrient	Database	Search Query	Results Count
**Calcium**	PubMed	"calcium"[Title/Abstract] AND ("diet*"[Title/Abstract] OR "intak*"[Title/Abstract]) AND ("dose-response"[Title/Abstract] OR "dose response"[Title/Abstract]) AND "meta analysis"[Publication Type] AND 2010:2026[Date - Publication]	30
	Scopus	(TITLE-ABS-KEY (calcium) AND TITLE-ABS-KEY (diet* OR intak*) AND TITLE-ABS-KEY ("dose response" OR dose-response) AND TITLE-ABS-KEY (meta-analysis)) AND PUBYEAR > 2010 AND PUBYEAR < 2026	103
	Web of Science	TS=(calcium) AND TS=(diet* OR intak*) AND TS=("dose-response" OR "dose response") AND TS=("meta-analysis") AND PY=(2010-2026)	122
**Iron**	PubMed	"iron"[Title/Abstract] AND ("diet*"[Title/Abstract] OR "intak*"[Title/Abstract]) AND ("dose-response"[All Fields] OR "dose response"[All Fields]) AND "meta analysis"[Publication Type] AND 2010:2026 [Date - Publication]	13
	Scopus	(TITLE-ABS-KEY (iron) AND TITLE-ABS-KEY (diet* OR intak*) AND TITLE-ABS-KEY ("dose response" OR dose-response) AND TITLE-ABS-KEY (meta-analysis)) AND PUBYEAR > 2010 AND PUBYEAR < 2026	34
	Web of Science	TS=(iron) AND TS=(diet* OR intak*) AND TS=("dose-response" OR "dose response") AND TS=("meta-analysis") AND PY=(2010-2026)	44
**Zinc**	PubMed	"zinc"[Title/Abstract] AND ("diet*"[Title/Abstract] OR "intak*"[Title/Abstract]) AND ("dose-response"[All Fields] OR "dose-response"[All Fields]) AND ("meta analysis"[Publication Type] AND 2010:2026[Date - Publication])	13
	Scopus	(TITLE-ABS-KEY (zinc) AND TITLE-ABS-KEY (diet* OR intak*) AND TITLE-ABS-KEY ("dose response" OR dose-response) AND TITLE-ABS-KEY (meta-analysis)) AND PUBYEAR > 2010 AND PUBYEAR < 2026	41
	Web of Science	TS=(zinc) AND TS=(diet* OR intak*) AND TS=("dose-response" OR "dose response") AND TS=("meta-analysis") AND PY=(2010-2026)	22
**Magnesium**	PubMed	"magnesium"[Title/Abstract] AND ("diet*"[Title/Abstract] OR "intak*"[Title/Abstract]) AND ("dose-response"[All Fields] OR "dose response"[All Fields]) AND ("meta analysis"[Publication Type] AND 2010:2026 [Date - Publication])	23
	Scopus	(TITLE-ABS-KEY (magnesium) AND TITLE-ABS-KEY (diet* OR intak*) AND TITLE-ABS-KEY ("dose response" OR dose-response) AND TITLE-ABS-KEY (meta-analysis)) AND PUBYEAR > 2010 AND PUBYEAR < 2026	49
	Web of Science	TS=(magnesium) AND TS=(diet* OR intak*) AND TS=("dose-response" OR "dose response") AND TS=("meta-analysis") AND PY=(2010-2026)	54
**Selenium**	PubMed	"selenium"[Title/Abstract] AND ("diet*"[Title/Abstract] OR "intak*"[Title/Abstract]) AND ("dose-response"[All Fields] OR "dose response"[All Fields]) AND ("meta analysis"[Publication Type] AND 2010:2026 [Date - Publication])	13
	Scopus	(TITLE-ABS-KEY (selenium) AND TITLE-ABS-KEY (diet* OR intak*) AND TITLE-ABS-KEY ("dose response" OR dose-response) AND TITLE-ABS-KEY (meta-analysis)) AND PUBYEAR > 2010 AND PUBYEAR < 2025	22
	Web of Science	TS=(selenium) AND TS=(diet* OR intak*) AND TS=("dose-response" OR "dose response") AND TS=("meta-analysis") AND PY=(2010-2026)	20
**Sodium**	PubMed	("sodium"[Title/Abstract] OR "salt"[Title/Abstract]) AND ("diet*"[Title/Abstract] OR "intak*"[Title/Abstract]) AND ("dose-response"[All Fields] OR "dose response"[All Fields]) AND ("meta analysis"[Publication Type] AND 2010:2026[Date - Publication])	24
	Scopus	(TITLE-ABS-KEY (sodium OR salt) AND TITLE-ABS-KEY (diet* OR intak*) AND TITLE-ABS-KEY ("dose response" OR dose-response) AND TITLE-ABS-KEY (meta-analysis)) AND PUBYEAR > 2010 AND PUBYEAR < 2026	69
	Web of Science	TS=(sodium OR salt) AND TS=(diet* OR intak*) AND TS=("dose-response" OR "dose response") AND TS=("meta-analysis") AND PY=(2010-2026)	49

**Copper**	PubMed	"copper"[Title/Abstract] AND ("diet*"[Title/Abstract] OR "intak*"[Title/Abstract]) AND ("dose-response"[All Fields] OR "dose response"[All Fields]) AND ("meta analysis"[Publication Type] AND 2010:2026 [Date - Publication])	2
	Scopus	(TITLE-ABS-KEY (copper) AND TITLE-ABS-KEY (diet* OR intak*) AND TITLE-ABS-KEY ("dose response" OR dose-response) AND TITLE-ABS-KEY (meta-analysis)) AND PUBYEAR > 2010 AND PUBYEAR < 2025	8
	Web of Science	TS=(copper) AND TS=(diet* OR intak*) AND TS=("dose-response" OR "dose response") AND TS=("meta-analysis") AND PY=(2010-2026)	4
**Vitamin B12**	PubMed	("vitamin B12"[Title/Abstract] OR "vitamin B 12"[Title/Abstract] OR "cobalamin"[Title/Abstract] OR "cyanocobalamin"[Title/Abstract] OR "methylcobalamin"[Title/Abstract]) AND ("diet*"[Title/Abstract] OR "intak*"[Title/Abstract]) AND ("dose-response"[All Fields] OR "dose response"[All Fields]) AND ("meta analysis"[Publication Type] AND 2010:2026[Date - Publication])	12
	Scopus	(TITLE-ABS-KEY ("vitamin B12" OR "vitamin B 12" OR cobalamin OR cyanocobalamin OR methylcobalamin*) AND TITLE-ABS-KEY (diet* OR intak*) AND TITLE-ABS-KEY ("dose response" OR dose-response) AND TITLE-ABS-KEY (meta-analysis)) AND PUBYEAR > 2010 AND PUBYEAR < 2026	27
	Web of Science	TS=("vitamin B12" OR "vitamin B 12" OR cobalamin OR cyanocobalamin OR methylcobalamin) AND TS=(diet* OR intak*) AND TS=("dose-response" OR "dose response") AND TS=("meta-analysis") AND PY=(2010-2026)	21
**Vitamin D3**	PubMed	("calciferol"[Title/Abstract] OR "cholecalciferol"[Title/Abstract] OR "vitamin d*"[Title/Abstract]) AND ("diet*"[Title/Abstract] OR "intak*"[Title/Abstract]) AND ("dose-response"[All Fields] OR "dose response"[All Fields]) AND ("meta analysis"[Publication Type] AND 2010:2026[Date - Publication])	34
	Scopus	(TITLE-ABS-KEY (calciferol OR cholecalciferol OR "vitamin D*") AND TITLE-ABS-KEY (diet* OR intak*) AND TITLE-ABS-KEY ("dose response" OR dose-response) AND TITLE-ABS-KEY (meta-analysis)) AND PUBYEAR > 2010 AND PUBYEAR < 2026	138
	Web of Science	TS=(calciferol OR cholecalciferol OR "vitamin D*") AND TS=(diet* OR intak*) AND TS=("dose-response" OR "dose response") AND TS=("meta-analysis") AND PY=(2010-2026)	103
**Thiamine**	PubMed	("thiamine*"[Title/Abstract] OR "vitamin B1"[Title/Abstract]) AND ("diet*"[Title/Abstract] OR "intak*"[Title/Abstract]) AND ("dose-response"[All Fields] OR "dose response"[All Fields]) AND ("meta analysis"[Publication Type] AND 2010:2026[Date - Publication])	0
	Scopus	(TITLE-ABS-KEY (thiamin* OR "vitamin B1") AND TITLE-ABS-KEY (diet* OR intak*) AND TITLE-ABS-KEY ("dose response" OR dose-response) AND TITLE-ABS-KEY (meta-analysis)) AND PUBYEAR > 2010 AND PUBYEAR < 2026	5
	Web of Science	TS=(thiamin* OR "vitamin B1") AND TS=(diet* OR intak*) AND TS=("dose-response" OR "dose response") AND TS=("meta-analysis") AND PY=(2010-2026)	2
**Niacin**	PubMed	("niacin*"[Title/Abstract] OR "vitamin B3"[Title/Abstract] OR "nicotinic acid"[Title/Abstract] OR "niacinamide"[Title/Abstract]) AND ("diet*"[Title/Abstract] OR "intak*"[Title/Abstract]) AND ("dose-response"[All Fields] OR "dose response"[All Fields]) AND ("meta analysis"[Publication Type] AND 2010:2026[Date - Publication])	1
	Scopus	(TITLE-ABS-KEY (niacin* OR "vitamin B3" OR "nicotinic acid" OR "niacinamide") AND TITLE-ABS-KEY (diet* OR intak*) AND TITLE-ABS-KEY ("dose response" OR dose-response) AND TITLE-ABS-KEY (meta-analysis)) AND PUBYEAR > 2010 AND PUBYEAR < 2026	8
	Web of Science	TS=(niacin* OR "vitamin B3" OR "nicotinic acid" OR "niacinamide") AND TS=(diet* OR intak*) AND TS=("dose-response" OR "dose response") AND TS=("meta-analysis") AND PY=(2010-2026)	1
**Fibre**	PubMed	("fibre"[Title/Abstract] OR "fiber"[Title/Abstract]) AND ("diet*"[Title/Abstract] OR "intak*"[Title/Abstract]) AND ("dose-response"[All Fields] OR "dose response"[All Fields]) AND ("meta analysis"[Publication Type] AND 2010:2026[Date - Publication])	48

	Scopus	(TITLE-ABS-KEY (fibre OR fiber) AND TITLE-ABS-KEY (diet* OR intak*) AND TITLE-ABS-KEY ("dose response" OR dose-response) AND TITLE-ABS-KEY (meta-analysis)) AND PUBYEAR > 2010 AND PUBYEAR < 2026	94
	Web of Science	TS=(fibre OR fiber) AND TS=(diet* OR intak*) AND TS=(“dose-response” OR “dose response”) AND TS=("meta-analysis") AND PY=(2010-2026)	149
**Saturated fatty acids**	PubMed	("saturated fatty acid*"[Title/Abstract] OR "saturated fat*"[Title/Abstract] OR "dietary fat*"[Title/Abstract]) AND ("diet*"[Title/Abstract] OR "intak*"[Title/Abstract]) AND ("dose-response"[All Fields] OR "dose response"[All Fields]) AND ("meta analysis"[Publication Type] AND 2010:2026[Date - Publication])	25
	Scopus	(TITLE-ABS-KEY ("saturated fatty acid*" OR "saturated fat*" OR "dietary fat*") AND TITLE-ABS-KEY (diet* OR intak*) AND TITLE-ABS-KEY ("dose response" OR dose-response) AND TITLE-ABS-KEY (meta-analysis)) AND PUBYEAR > 2010 AND PUBYEAR < 2026	58
	Web of Science	TS=("saturated fatty acid*" OR "saturated fat*" OR "dietary fat*") AND TS=(diet* OR intak*) AND TS=(“dose-response” OR “dose response”) AND TS=("meta-analysis") AND PY=(2010-2026)	66
**n-3 fatty acids**	PubMed	("n-3 fatty acids"[Title/Abstract] OR "omega-3"[Title/Abstract] OR "essential fatty acids"[Title/Abstract] OR "n-3 PUFA"[Title/Abstract] OR "alpha-linolenic acid"[Title/Abstract]) AND ("diet*"[Title/Abstract] OR "intak*"[Title/Abstract]) AND ("dose-response"[All Fields] OR "dose response"[All Fields]) AND ("meta analysis"[Publication Type] AND 2010:2026[Date - Publication])	45
	Scopus	(TITLE-ABS-KEY ("n-3 fatty acids" OR "omega-3" OR "essential fatty acids" OR "n-3 PUFA" OR "alpha-linolenic acid") AND TITLE-ABS-KEY (diet* OR intak*) AND TITLE-ABS-KEY ("dose response" OR dose-response) AND TITLE-ABS-KEY (meta-analysis)) AND PUBYEAR > 2010 AND PUBYEAR < 2026	118
	Web of Science	TS=("n-3 fatty acids" OR "omega-3" OR "essential fatty acids" OR "n-3 PUFA" OR "alpha-linolenic acid") AND TS=(diet* OR intak*) AND TS=(“dose-response” OR “dose response”) AND TS=("meta-analysis") AND PY=(2010-2026)	91
**n-6 fatty acids**	PubMed	("n-6 fatty acids"[Title/Abstract] OR "omega-6"[Title/Abstract] OR "essential fatty acids"[Title/Abstract] OR "n-6 PUFA"[Title/Abstract] OR "linoleic acid"[Title/Abstract]) AND ("diet*"[Title/Abstract] OR "intak*"[Title/Abstract]) AND ("dose-response"[All Fields] OR "dose response"[All Fields]) AND ("meta analysis"[Publication Type] AND 2010:2026[Date - Publication])	14
	Scopus	(TITLE-ABS-KEY ("n-6 fatty acids" OR "omega-6" OR "essential fatty acids" OR "n-6 PUFA" OR "linoleic acid") AND TITLE-ABS-KEY (diet* OR intak*) AND TITLE-ABS-KEY ("dose response" OR dose-response) AND TITLE-ABS-KEY (meta-analysis)) AND PUBYEAR > 2010 AND PUBYEAR < 2026	35
	Web of Science	TS=("n-6 fatty acids" OR "omega-6" OR "essential fatty acids" OR "n-6 PUFA" OR "linoleic acid") AND TS=(diet* OR intak*) AND TS=(“dose-response” OR “dose response”) AND TS=("meta-analysis") AND PY=(2010-2026)	25
**MUFA**	PubMed	("MUFA*"[Title/Abstract] OR "oleic acid"[Title/Abstract] OR "monounsaturated fat*"[Title/Abstract]) AND ("diet*"[Title/Abstract] OR "intak*"[Title/Abstract]) AND ("dose-response"[All Fields] OR "dose response"[All Fields]) AND ("meta analysis"[Publication Type] AND 2010:2026[Date - Publication])	16
	Scopus	(TITLE-ABS-KEY (mufa* OR "oleic acid" OR "monounsaturated fat*") AND TITLE-ABS-KEY (diet* OR intak*) AND TITLE-ABS-KEY ("dose response" OR dose-response) AND TITLE-ABS-KEY (meta-analysis)) AND PUBYEAR > 2010 AND PUBYEAR < 2026	28
	Web of Science	TS=(mufa* OR "oleic acid" OR "monounsaturated fat*") AND TS=(diet* OR intak*) AND TS=(“dose-response” OR “dose response”) AND TS=("meta-analysis") AND PY=(2010-2026)	24

Supplementary Table S2. Result counts for each included nutrient and database.

Nutrient	PubMed	Scopus	Web of Science	Total
**Calcium**	30	103	122	255
**Iron**	13	34	44	91
**Zinc**	13	41	22	76
**Magnesium**	23	49	54	126
**Selenium**	13	22	20	55
**Sodium**	24	69	49	142
**Copper**	2	8	4	14
**Vitamin B12**	12	27	21	60
**Vitamin D3**	34	138	103	275
**Thiamine**	0	5	2	7
**Niacin**	1	8	1	10
**Fibre**	48	94	149	291
**Saturated fatty acids**	25	58	66	149
**n-3 fatty acids**	45	118	91	254
**n-6 fatty acids**	14	35	25	74
**MUFA**	16	28	24	68
**Total**	313	837	797	1947

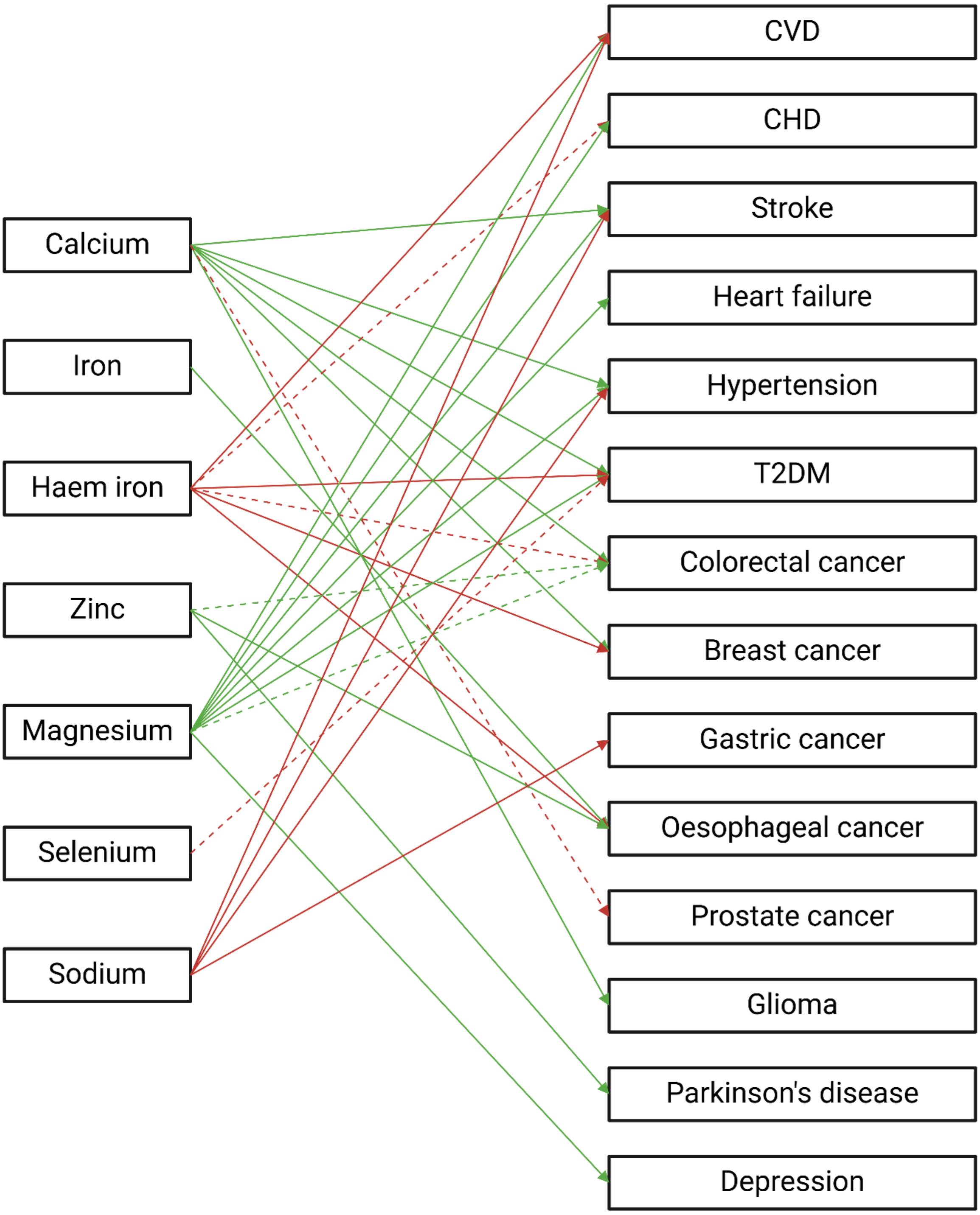

Supplementary Figure S1. Health outcomes associated with each of the selected nutrients, with solid lines indicating associations from studies with low risk of bias, and dashed lines indicating associations from studies with high risk of bias. Red lines indicate potential risks, and green lines indicate potential benefits.

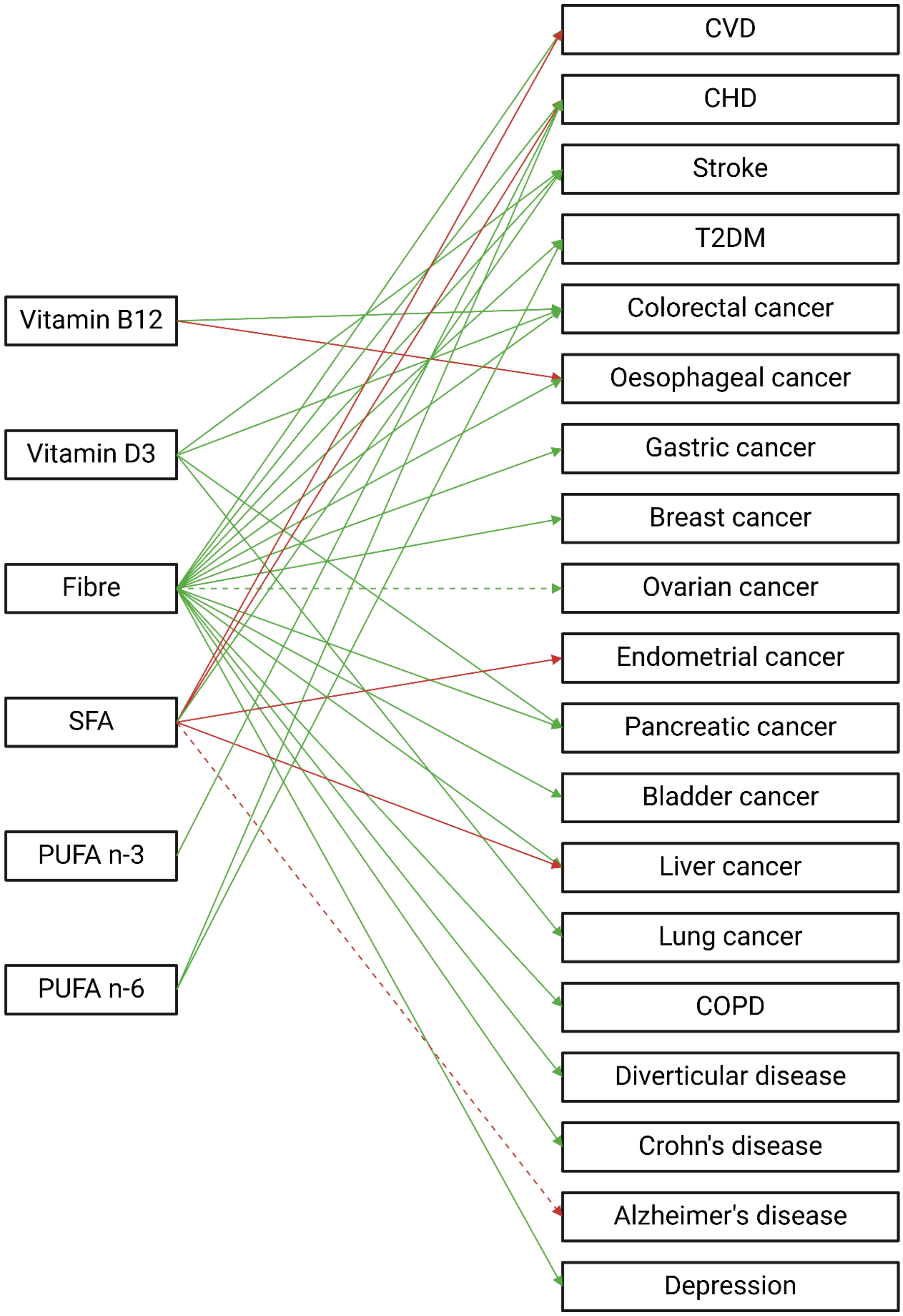

Supplementary Figure S2. Health outcomes associated with each of the selected nutrients, with solid lines indicating associations from studies with low risk of bias, and dashed lines indicating associations from studies with high risk of bias. Red lines indicate potential risks, and green lines indicate potential benefits.
